# Recognizing Synchronous Multiple Primary Lung Cancer: A Case Report

**DOI:** 10.7759/cureus.85729

**Published:** 2025-06-10

**Authors:** Noah Gordon, Danielle Gratza, Nahren Asado, Wissam Raad, Abdul Alraiyes

**Affiliations:** 1 Internal Medicine, Advocate Lutheran General Hospital, Park Ridge, USA; 2 Pathology, Advocate Lutheran General Hospital, Park Ridge, USA; 3 Thoracic Surgery, Advocate Lutheran General Hospital, Park Ridge, USA; 4 Interventional Pulmonology, Advocate Lutheran General Hospital, Park Ridge, USA

**Keywords:** cigarette smoking, field cancerization, incidental radiological finding, lung cancer staging, multi-disciplinary teams, non small cell lung cancer, pleural nodule, pulmonary carcinoid tumor, synchronous primary cancers

## Abstract

Pulmonary nodules are frequently identified on radiographic evaluation, often due to lung cancer screening with low-dose computed tomography, an increasingly utilized technology in high-risk patients, or incidentally discovered with alternative imaging modalities. Although most nodules are benign, lung cancer remains difficult to treat, particularly in its late stages, which are more prone to metastasis. We present a case of a 71-year-old female, a former smoker, who was evaluated in the emergency department for chronic cough and dyspnea. On evaluation, computed tomography pulmonary angiography incidentally revealed three distinct pulmonary nodules. Following evaluation by our multidisciplinary team, robot-assisted bronchoscopy (RAB) was performed, revealing three distinct primary malignancies: well-differentiated adenocarcinoma with KRAS mutation, poorly differentiated adenocarcinoma with EGFR mutation, and a well-differentiated carcinoid tumor. The patient underwent staged surgical resections for curative intent. This case underscores the limitations of strict adherence to existing pulmonary nodule guidelines, such as those from the Fleischner Society, which, in this instance, could have led to inaccurate staging and palliative systemic therapy. It also highlights the critical value of considering biopsy of multiple nodules, now increasingly feasible with advanced technologies such as RAB. Early and accurate characterization of each lesion allowed for appropriate staging and individualized, potentially curative treatment.

## Introduction

Synchronous multiple primary lung cancer (SMPLC) refers to the simultaneous diagnosis of two or more distinct cancerous lesions rather than metastasis from a single primary site. This phenomenon is estimated to occur in approximately 2% of patients diagnosed with lung cancer [1]. Identifying this subset of patients is vital to ensure that an accurate diagnosis is made; otherwise, treatment may be ineffective against one or more nodule types. Although the underlying pathogenesis is incompletely understood, carcinogen exposure is able to create a large area of genetic alteration underpinning the risk of dysplasia - a concept known as field cancerization. This notion is supported by increased tobacco exposure and genetic mutations such as p53 and KRAS being observed more in patients with SMPLC when compared to patients with a single tumor [2]. We report the case of a 71-year-old female in whom three distinct primary lung cancers were identified: carcinoid tumor, adenocarcinoma with KRAS mutation, and adenocarcinoma with EGFR mutation.

## Case presentation

**Figure 1:**
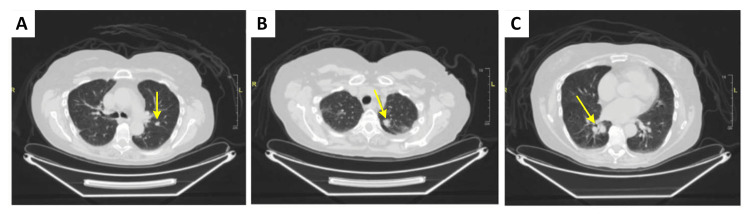
Axial chest CT images demonstrating three distinct pulmonary nodules biopsied during a single robot-assisted bronchoscopy procedure. A) Left upper lobe nodule #1, diagnosed as a typical carcinoid tumor (well-differentiated neuroendocrine tumor, grade 1). B) Left upper lobe nodule #2, diagnosed as well-differentiated pulmonary adenocarcinoma with a predominant acinar pattern. C) Right lower lobe nodule, diagnosed as poorly differentiated pulmonary adenocarcinoma with a mostly solid pattern. Yellow arrows indicate the location of each nodule.

A 71-year-old female with a past medical history of post-operative hypothyroidism following thyroidectomy for non-toxic multinodular goiter presented to the emergency department (ED) for worsening dyspnea and mid-sternal chest pain in the setting of a 10-week chronic, non-productive cough. At the onset of symptoms, she described having a cold-like prodrome including fever, chills, and congestion. Although her cold-like symptoms resolved, her cough persisted. Social history was significant for a 15-pack-year smoking history, and a family history was notable for lung cancer. Given the concern for pulmonary embolism (PE) in the setting of acute-onset dyspnea, a computed tomography pulmonary angiogram (CTPA) was performed. Though a PE was not discovered, three incidental pulmonary nodules were identified: a 15 mm x 10 mm spiculated noncalcified nodule in the left upper lobe (LUL), an 11 mm x 9 mm noncalcified nodule also in the LUL, and an 18 mm x 16 mm spiculated noncalcified nodule in the right lower lobe (RLL) (Figure 1). Further workup in the ED was otherwise unremarkable, and the patient was discharged home with plans to follow up with pulmonology outpatient.

**Figure 2:**
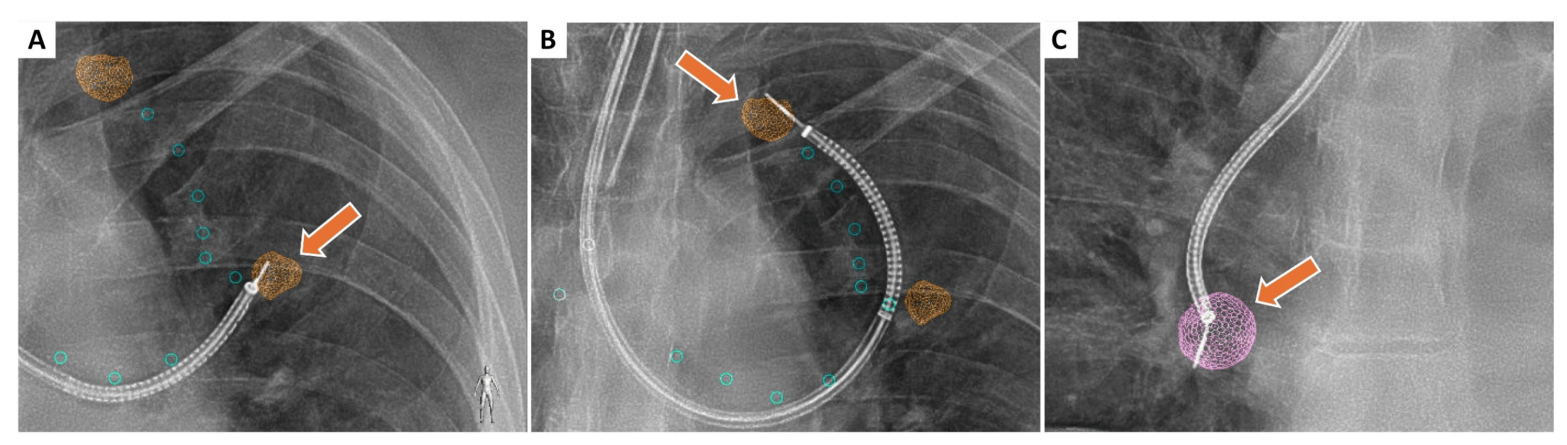
Fluoroscopic images obtained during robot-assisted bronchoscopy with cone-beam CT and augmented fluoroscopy. Orange arrows highlight the targeted pulmonary nodules in both lungs: (A) typical carcinoid, (B) well-differentiated adenocarcinoma, and (C) poorly differentiated adenocarcinoma. The orange and purple overlays represent the augmented fluoroscopy rendering of the nodule location in the left upper lobe and right lower lobe, and radial endobronchial ultrasound confirmation of lesion position prior to transbronchial biopsy.

The following week, a positron emission tomography (PET) scan was performed, which demonstrated multiple small radioactive nodules within bilateral lung fields without evidence of metastatic disease. The standardized uptake value (SUV) on PET was 2.07 for the 15 mm x 10 mm LUL nodule, 2.24 for the 11 mm x 9 mm LUL nodule, and 1.75 for the 18 mm x 16 mm RLL nodule. Although SUV values below 2.5 are generally suggestive of a benign etiology, PET imaging alone is not sufficient to make that determination. Image findings were discussed with a multidisciplinary tumor board that included teams from thoracic surgery, medical oncology, pulmonary radiology, interventional pulmonology, and pathology. In the interim, she was prescribed a course of azithromycin to decrease airway inflammation in the context of possible infectious bronchitis. A repeat chest computed tomography (CT) scan performed six weeks later showed no change in lung nodule size, and due to this lack of growth and small size, general radiology recommended repeat surveillance imaging in 12 months. However, due to nodule spiculation, lack of interval size reduction, high-risk social history including a 15-pack-year cigarette smoking history, and procedural feasibility, the multidisciplinary tumor board decided to move forward with biopsying all three pulmonary nodules. To successfully obtain biopsies, the patient underwent an ion robot-assisted bronchoscopy with cone beam CT with augmented fluoroscopy and radial endoscopic ultrasound position confirmation (Figure 2). A transbronchial biopsy of the RLL lesion and two LUL lesions were obtained. Biopsy specimens were evaluated intraoperatively by a pathologist for adequacy and preserved for review.

**Figure 3:**
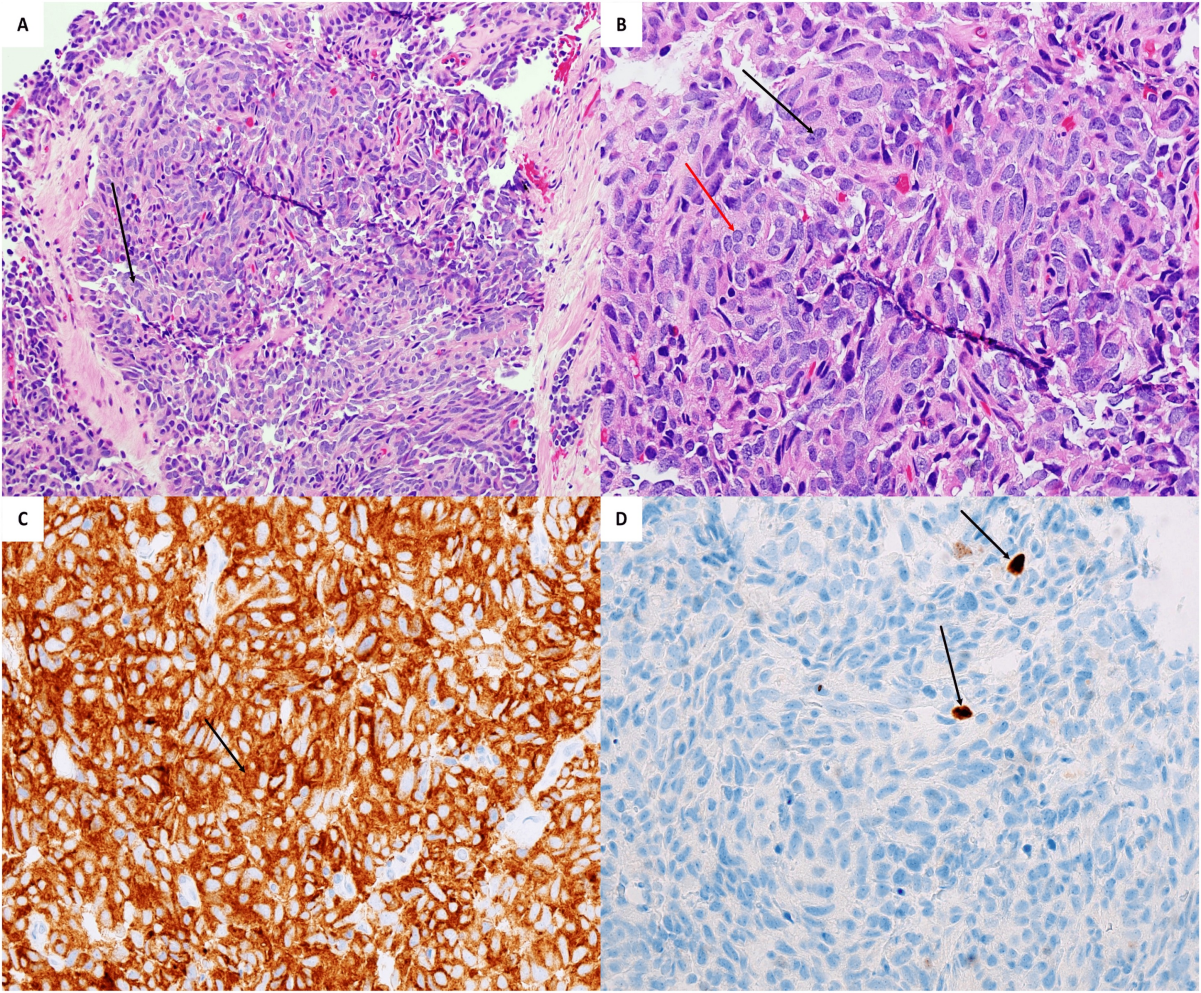
Left upper lobe nodule identified as typical carcinoid tumor, grade 1, on transbronchial biopsy. A) Large nests of uniform tumor cells (black arrow) and small vascular spaces. B) Elongated (back arrow) and round (red arrow) nuclei with finely stippled chromatin pattern. C) Synaptophysin immunohistochemical staining shows positive cytoplasmic granular staining (black arrow). D) Ki-67 immunohistochemical staining of approximately 1% of tumor cells (black arrows).

**Figure 4:**
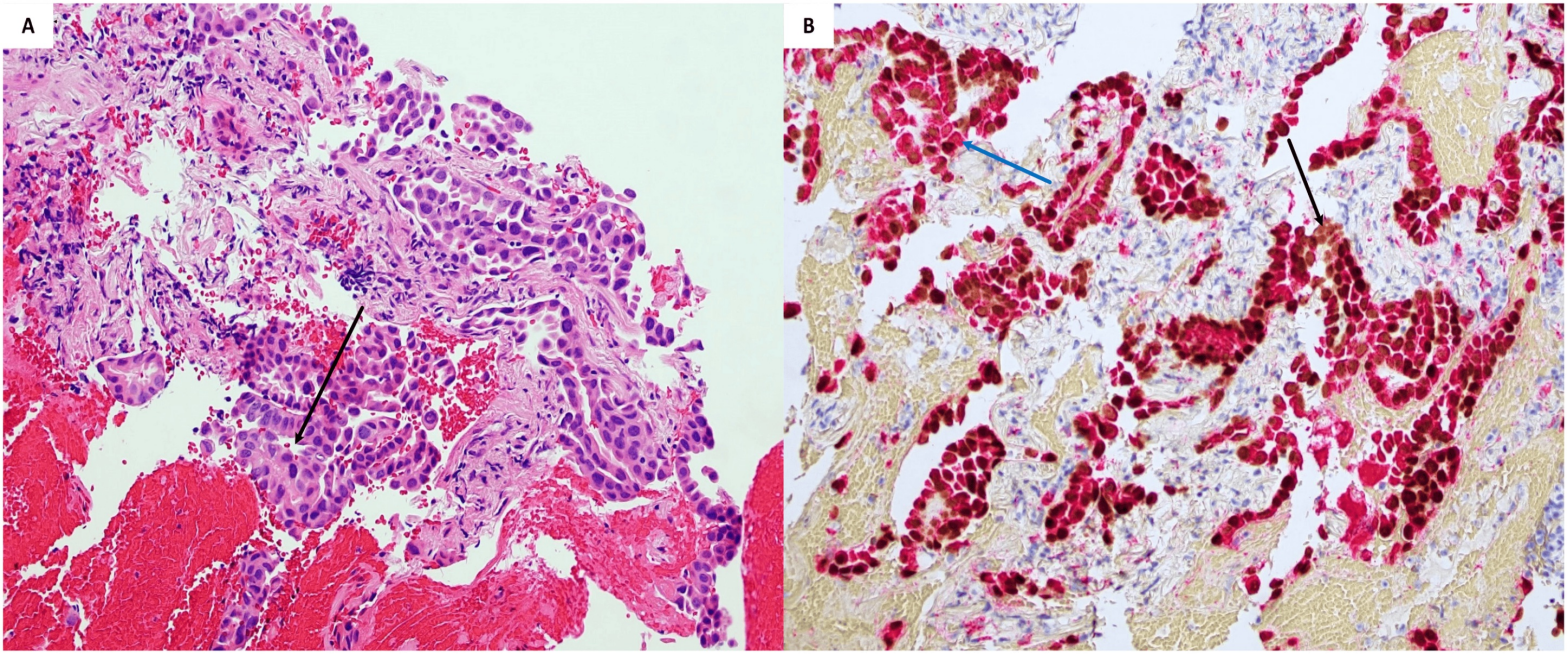
Left upper lobe nodule identified as well-differentiated adenocarcinoma with a predominant acinar pattern on transbronchial biopsy. A) Malignant adenocarcinoma cells forming glands (black arrow) and growing along alveolar septa. B) Positive staining for TTF1 (black arrow) and Napsin A (blue arrow). TTF1, thyroid transcription factor 1

**Figure 5:**
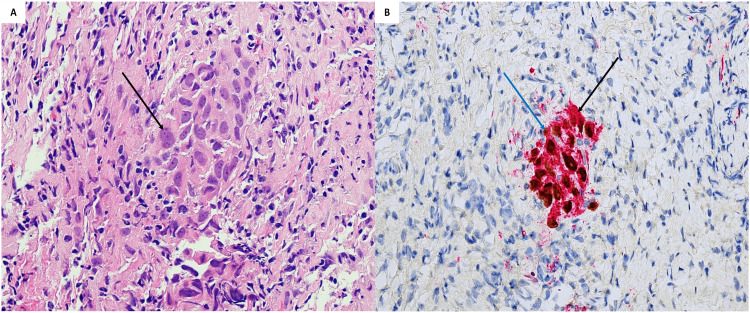
Right lower lobe nodule biopsy identified as poorly differentiated pulmonary adenocarcinoma with a predominant solid pattern on transbronchial biopsy. A) Cluster of solid cells with pleomorphic nuclei (black arrow). B) Immunohistochemical staining positive for TTF1 (blue arrow) and Napsin A (black arrow). TTF1, thyroid transcription factor 1

Pathology demonstrated three distinct cancerous morphologies consistent with SMPLC (Figures 3-5). The first LUL lesion showed well-differentiated carcinoid tumor stage IA2 (cT1bN0M0), according to the American Joint Committee on Cancer (AJCC) lung cancer staging, 8th edition. The second LUL nodule showed well-differentiated adenocarcinoma with predominant acinar pattern stage IA2 (cT1bN0M0) with KRAS mutation. The RLL nodule showed poorly differentiated adenocarcinoma with a mostly solid pattern stage IA3 (pT1cN0M0) with an EGFR mutation.

Due to the RLL nodule being poorly differentiated and larger in size, the multidisciplinary tumor board recommended prioritizing curative resection of this nodule first. Thus, a right lower lobectomy with mediastinal node dissection was performed without immediate complications. After a two-month recovery, the LUL adenocarcinoma lesion was then addressed via left apicoposterior segmentectomy with lymph node dissection. A biopsy of this resected segment identified two foci measuring 1.9 cm and 1.2 cm that tested positive for invasive adenocarcinoma, which increased in staging from stage IA2 (cT1bN0M0) to stage IIB (cT3N0M0). Due to relatively low staging overall, the patient was not started on any adjuvant chemotherapy. The LUL carcinoid nodule was not resected, and repeat surveillance imaging showed significant improvement in nodule size.

## Discussion

Pulmonary nodules are detected in approximately 30% of chest CT scans; fortunately, of the nodules identified, around 95% of them are found to be benign in pathology [3]. Although most nodules are benign, lung cancer remains the leading cause of cancer death worldwide [4]. Therefore, the care management decisions that follow nodule identification represent a crucial moment in patient care.

Broadly, on CT imaging, there are several characteristics that may suggest that a nodule has a malignant etiology, such as spiculation, lobulation, and convergence of vasculature toward a nodule [5]. Similarly, nodule size is also correlated with a likelihood of malignancy, with nodules greater than 20 mm having been shown to have a higher propensity for malignancy [5, 6]. Despite none of the nodules in our patient meeting this size threshold, two of them were spiculated.

One established guideline to help the clinician determine appropriate management is the “Fleischner Society Guidelines for Incidental Pulmonary Nodules.” When applied to our case, the presence of multiple solid nodules measuring >8 mm indicated a repeat chest CT in three to six months [7]. This follow-up interval is designed to balance the risk of false-positives with the knowledge that malignant nodules typically double in size within approximately 400 days - so if cancerous, interval growth should be detectable in three to six months [8]. Additionally, the Fleischner Society guidelines only prioritize obtaining biopsy of the most suspicious nodule [7]. In our case, this would have been the RLL nodule due to it being the largest and spiculated in appearance. However, if we rigidly adhered to these guidelines, our patient would have been inaccurately diagnosed with metastatic stage IVa adenocarcinoma and subjected to palliative systemic chemotherapy rather than surgical intervention with curative intent.

The lungs are a common site of metastasis of cancer originating from a different organ, but they are also the most common site of metastasis from primary lung cancer [9]. Despite this fitting the narrative of a stage IV malignancy, the presence of spiculated nodules or air bronchograms is more common in SMPLC rather than true metastatic spread [10,11]. This observation in combination with the patient's risk factors played a critical role in deviating from the Fleischner Society Guidelines and quickly advancing to interventional diagnostic tools.

There is a myriad of environmental exposures known to be associated with an increased risk of developing pulmonary malignancy, such as exposures to asbestosis, radon, air pollution, and either first- or secondhand cigarette smoke [12,13]. Notably, many types of exposures are inhalants that will contact a broad area of lung tissue. This introduces the concept of field effect, where genetic mutations are secondary to interaction of a broad environmental insult, such as smoking in our patient [14]. Genetic susceptibility varies among individuals, which helps explain why some people develop malignancy even after minimal exposure to carcinogens [15]. Therefore, in the context of multiple pulmonary nodules, exposure to a known carcinogen or a strong family history of pulmonary malignancy should prompt clinicians to consider the possibility of SMPLC.

Since every patient is unique, strictly following recommendations such as the Fleischner Society Guidelines for Incidental Pulmonary Nodules can lead to misdiagnosis, as we demonstrated, and, therefore, mistreatment of patients. Often, general practitioners and general radiologists lack the specialized training to determine the best next step after pulmonary nodules are seen on imaging [16]. In our case, a multidisciplinary team approach was implemented shortly after discovering pulmonary lesions on CT scan. A tumor board meeting was held with multidisciplinary teams from interventional pulmonology, thoracic surgery, medical oncology, pulmonary radiology, and pathology. Given the patient’s 15-pack-year smoking history, family history of lung cancer, and the radiographic variability in size, morphology, and location of the nodules, the multidisciplinary team recommended biopsy of all three pulmonary nodules. The use of robot-assisted bronchoscopy - in combination with cone-beam CT, augmented fluoroscopy, and radial endobronchial ultrasound - allowed for precise localization and sequential sampling of all three lesions during a single procedure. This comprehensive approach minimized procedural risk, expedited diagnosis, and ensured accurate staging by confirming the histology of each nodule independently. For our patient, this resulted in a diagnosis of three different primary cancers, well-differentiated acinar-predominant adenocarcinoma, poorly differentiated solid-predominant adenocarcinoma, and a well-differentiated carcinoid tumor. Notably, typical carcinoid tumors have no known relationship to smoking [17]. Thus, in our patient, this nodule may be completely unrelated to her smoking history unlike her other two cancerous lesions.

Following pathology results, the case was discussed with multidisciplinary teams again and the decision for surgical resection was made. Collaborating with different specialties allows each to share their own individual expertise and knowledge on lung cancer. The management of one patient may not always be the same for another patient. Therefore, this integrative approach ensures that the best clinical decision-making is applied to each patient and helps ensure adequate patient follow-up. Some institutions have adopted comprehensive lung nodule management programs that begin with a referral to a lung nodule navigator once a suspicious nodule is identified. The navigator serves as the central point of coordination, bringing together a multidisciplinary team that may include pulmonologists, radiologists, thoracic surgeons, oncologists, and primary care providers. This role is essential in streamlining the evaluation process, ensuring timely workup, and reducing delays in care. Navigators help standardize workflows, facilitate risk-based triage, and ensure adherence to evidence-based guidelines. In addition to logistical coordination, they provide education and support to patients, improving engagement and compliance throughout the diagnostic and treatment journey. Their involvement is critical in reducing care fragmentation and ensuring that each case receives thorough, individualized attention from detection through management [18].

## Conclusions

Recognizing SMPLC is critical to ensuring accurate diagnosis and appropriate management. Misclassifying distinct primary lesions as metastatic disease can lead to unnecessary systemic therapy and missed opportunities for curative treatment. Clinicians should remain vigilant when evaluating patients with multiple pulmonary nodules, particularly those with risk factors such as smoking history, family predisposition, or radiographic features suggestive of malignancy. A multidisciplinary approach that incorporates advanced diagnostic technologies, such as robot-assisted bronchoscopy with cone-beam CT and augmented fluoroscopy, enables comprehensive and simultaneous biopsy of multiple nodules during a single procedure. This strategy supports accurate staging and individualized treatment planning. Early involvement of lung nodule navigators and integration of structured care pathways can further reduce delays, improve coordination, and enhance patient outcomes in complex lung cancer presentations.
